# Supplementary material for: Understanding and managing new risks on the Nile with the Grand Ethiopian Renaissance Dam
Source: Nat Commun. 2020 Oct 16;11:5222. doi: 10.1038/s41467-020-19089-x (PMC7567800; doi:10.1038/s41467-020-19089-x)
Supplement: Supplementary file 1 — Supplementary Information [file 41467_2020_19089_MOESM1_ESM.pdf]

Supplemental Material

Understanding and Managing New Risks on the Nile with the Grand Ethiopian Renaissance Dam

Contents:

Supplementary Note 1: Relation to the existing Nile systems analysis literature

Supplementary Note 2: Additional contextual information

Supplementary Note 3: Further details for Era 1 - Filling the GERD

Supplementary Note 4: Evaporation considerations

Supplementary Note 5: Statistical trace selections

Supplementary Note 6: Sensitivity analysis of existing uses

Supplementary Tables:

Supplementary Table 1. Key elevations and storage values of modeled reservoirs

Supplementary Table 2. Key modeling assumptions for the analysis of three stylized eras

Supplementary Table 3: Naturalized average annual flow at Aswan and Years of Hydrology

Supplementary Table 4. Effects of the GERD filling across low, average and high hydrologic scenarios

Supplementary Table 5. Effects of the GERD during *new normal* operations under a selection of average hydrologic conditions

Supplementary Table 6. Effects of the GERD during the onset and recovery from drought

<u>Supplementary Note 1: Relation to the existing Nile systems analysis literature</u>

The existing systems analysis literature on the Nile focuses largely on methodological issues in the simulation and optimization of multi-reservoir systems. Some papers include stochastic flow sequences [1-4] and various climate scenarios [5-10]. Most authors use simplified representations of the

Nile infrastructure in order to illustrate new methods. Several studies have sought to quantify the value of consequences in economic terms [1,5,10-13]. Although such quantification of economic consequences is useful when applying formal decision analysis, most presume some degree of idealized or optimal operations. In actual negotiations on contested rivers, decision makers are typically more interested in directly observable consequences of proposed agreements and plans (e.g., reservoir releases and levels).

We have been a part of and learned from this systems literature on the Nile. As a result, in this paper we distill the salient aspects of the system behavior and reflect these aspects in a small number of selected flow sequences. In contrast to the existing literature, the analysis in this paper focuses on the actual operational characteristics and observable consequences (e.g. reservoirs releases and levels) of the Eastern Nile system, which we believe will make our results more credible to decision makers. Although most of the papers in this literature describe the transboundary context and acknowledge the differing objectives of the various Nile riparians, none uses a narrative methodology to assist decision-makers to interpret modeling results.

We have used historic flow series as part of a narrative methodology to complement rather than supersede stochastic simulations and associated uncertainty methodologies that involve more exhaustive or probabilistic analysis of extreme events [4,14]. These stochastic methods are attractive for exploring the inevitable variability and uncertainty in river flows, and have been found to lead to more efficient water resource management plans [15]. However, we question their applicability in highly contested situations where actors have conflicting understandings and where trust is low. The appropriate use of combinations of methods will depend on context, but we emphasize that the use of historical, narrative methodologies such as presented in this paper should not be viewed as a methodologically inferior approach.

Supplementary Note 2: Additional contextual information

The Nile is the longest river in the world at 6695 km. It drains an area of 3.18 million km^2 located in 11 nations (Burundi, Democratic Republic of Congo, Egypt, Eritrea, Ethiopia, Kenya, Rwanda, South Sudan, Sudan, Tanzania and Uganda) (Figure 1). The majority of the flow comes from two major tributaries, the Blue Nile and the White Nile. The Blue Nile is a highly seasonal river that emerges from Lake Tana in the highlands of Ethiopia, and descends through a deep gorge before crossing into Sudan and flowing north to Khartoum, the capital of Sudan. The second major tributary emerges from Lake Victoria as the Victoria Nile at Jinja in Uganda, and flows northwards through Uganda and into the Sudd wetlands of South Sudan. Evaporation there reduces flow significantly, even after it joins with the Bahr el Ghazal and the Sobat rivers to form the White Nile. The Main Nile begins downstream of the confluence of the White and Blue Nile tributaries in Khartoum and continues northward through the Nubian desert before entering the reservoir behind the HAD. The seasonal Atbara River joins the Main Nile in northern Sudan. The HAD controls water releases that flow through the river channel in Egypt towards Cairo and the Mediterranean Sea.

The High Aswan Dam (HAD) was completed in 1970 (Supplementary Table 1). It has a minimum operating level of 147 masl and corresponding dead storage of 31.6 bcm. The top of active storage zone is as 175 masl and provides an active storage volume of 87.2 bcm. Approximately 7 bcm of sediment has accumulated in the reservoir since construction, with 46% deposited in the active storage zone. From 175 to 182 masl is the flood zone, which can hold up to 39.8 bcm. The flood management policy indicates that the pool elevation should be at or below 175 masl by August 1 of each year to accommodate the incoming flood season and minimize the risk of emergency releases that might negatively affect the islands and archeological sites directly downstream in the town of Aswan. The HAD operations also include a Drought Management Policy (DMP) where the withdrawals from the HAD Reservoir are reduced by 5% if the storage (elevation) of the reservoir

falls below 60 bcm (159.4 m), 10% if the reservoir falls below 55 bcm (157.6 m), and 15% if the reservoir falls below 50 bcm (155.7 m).

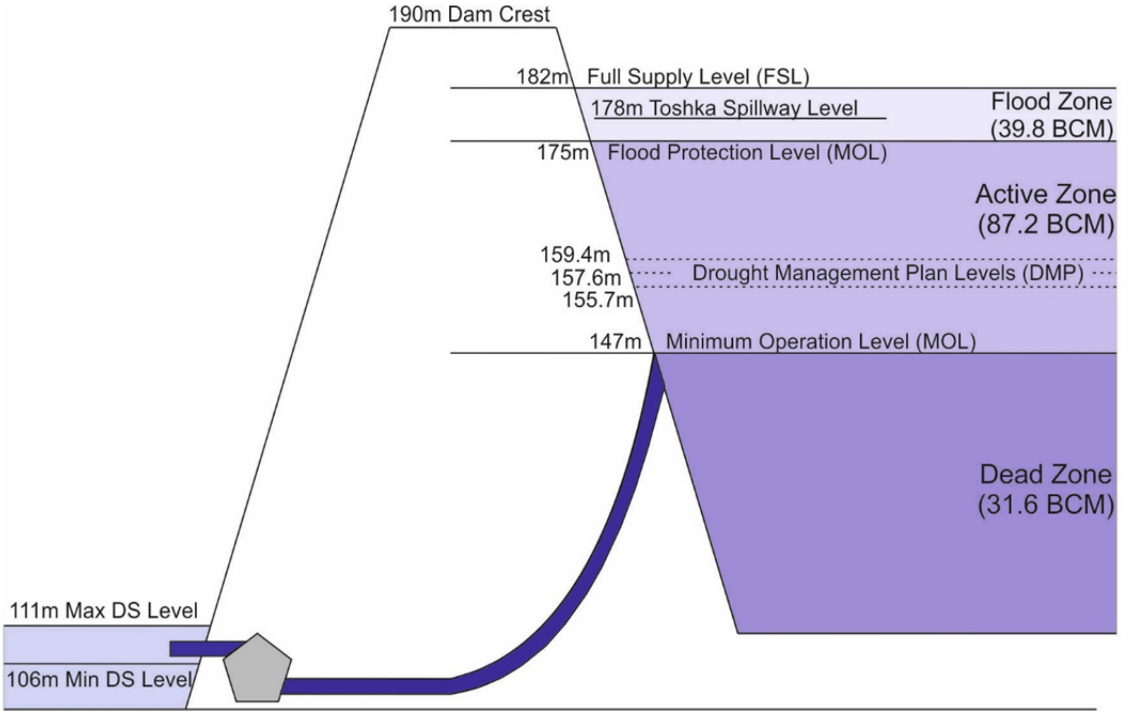

Supplementary Figure 1 – A side profile of the HAD showing different operating zones and their volumes and key operational elevations

Other existing infrastructure on the Blue Nile includes the Roseries Dam (5.9 bcm) and Sennar Dam (0.6 bcm) in Sudan. The largest reservoir in Sudan is created by the Merowe Dam on the Main Nile, with an active storage volume 8.1 bcm. Details of these dams and other smaller Sudanese and Ethiopian infrastructure are provided in Supplementary Table 1. Reservoir operations for each of these structures are described in previous publications [16].

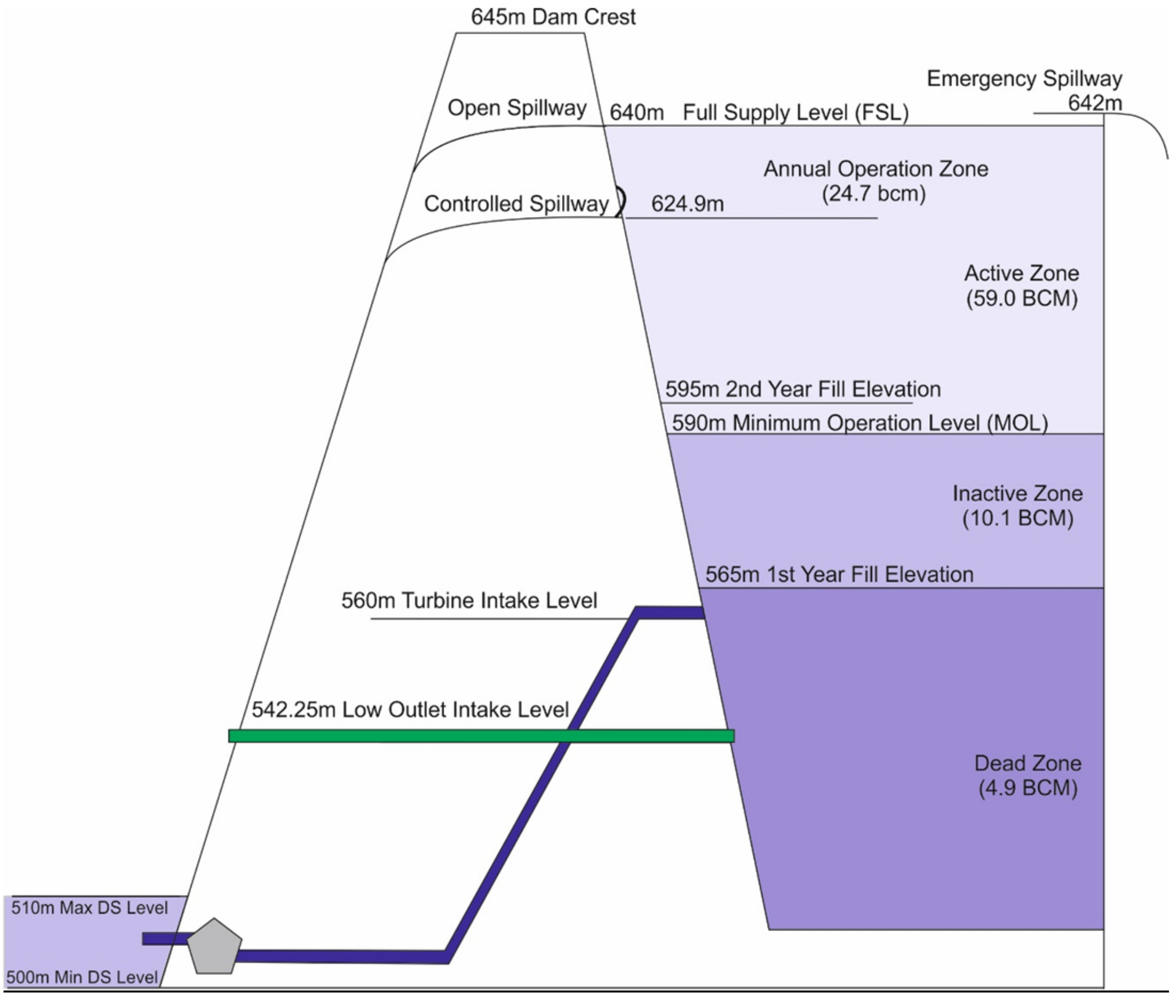

Supplementary Figure 2 – A side profile of the GERD showing different operating zones and their volumes and key operational elevations

<u>Supplementary Note 3: Further details for Era 1 - Filling the GERD</u>

Filling the GERD is expected to happen in three stages: (i) retain 4.9 bcm to test 2 low-head turbines at 565m in filling year 1, (ii) retain approximately 13.5 bcm to test the remainder of the turbines at 595 masl during filling year 2, and (iii) gradually fill the remaining active storage space to raise the reservoir to 625 masl at the beginning of a flood season (or 640 masl at the end of a flood season) over some negotiated duration of time.

Although the low-head turbines could in principle allow the GERD to be drawn down to 565 masl, recent statements from the ongoing negotiations suggest that 595 masl would be the lowest permissible level, therefore we consider the initial 18.4 bcm as a one-time 'cost' to fill the reservoir, which is roughly deducted from the inflows to the HAD Reservoir. More precisely however, a portion of this 18.4 bcm would never have reached the HAD Reservoir due to evaporation and seepage between the GERD and the HAD Reservoir. In addition, some of this water could in theory be consumed by water users in Sudan, though the consumption lost in Sudan is likely to be minor given existing water uses. Thus, the correspondence between the water held in storage in the GERD and the reduction in storage in the HAD Reservoir is somewhat more than one-to-one. In other words, the net storage reduction in the HAD Reservoir will be somewhat less than 18.4 bcm.

The downstream consequences of filling the GERD will depend on several natural and human-controlled factors. The first important factor will be the magnitude of flows throughout the basin during the filling period. High flows that occur in the Blue Nile above the GERD location are likely to shorten the period required for filling the GERD Reservoir, but this duration will also depend on operational decisions for the GERD as described below. Even if higher than average flows occur during filling below the GERD location on the Blue Nile or in the other tributaries, the level of the HAD Reservoir is still likely to fall due to the water captured by the GERD Reservoir. Our results indicate however that under wet conditions, Egypt likely will not need to reduce releases to avoid the HAD Reservoir falling to 147 masl. Some losses of hydropower in Egypt are still likely to occur however due to the lower average operational levels in the HAD Reservoir. Higher basin flows during and immediately following the filling period would accelerate the HAD Reservoir recovery.

Conversely, in the event of below-average basin flows during GERD Reservoir filling, the pool elevation in the HAD can be expected to decline more abruptly, and the extent of decline and the

potential impact on Egypt will depend on the operation of both dams. Under significant drought conditions, Egypt would prefer that Ethiopia not retain water in the GERD Reservoir and pass the entire (low) flow to downstream riparians, and possibly release stored water in the GERD Reservoir if needed. Regardless of how the GERD is operated, it is important to emphasize that only about 53% of the annual flows reaching the HAD Reservoir passes through the location of the GERD on average [17]. The remainder of the flow comes from the White Nile (nearly half from the Sobat – the Baro in Ethiopia) and several other tributaries, e.g., the Rahad and Dinder, and the Atbara (all from Ethiopia), which all meet the Nile downstream of the GERD site.

A second factor affecting the downstream impacts of the GERD during the filling process is the operational decisions for the GERD itself. This the primary focus of negotiations between the three countries. The less water that is released from the GERD during the filling process, the faster the retained water will accumulate in GERD Reservoir, but this will also result in more abrupt declines in the HAD Reservoir. Egypt would prefer more water to be released from the GERD throughout the filling period, resulting in a longer time required to fill the GERD Reservoir and a more gradual decline in the elevation of the HAD Reservoir. This would benefit Egypt by allowing more water under their immediate control to meet downstream needs and minimizing losses of power generation due to higher head at the HAD. However, a slower filling also comes at a cost of lower power generation from the GERD, as well as greater evaporation loss from the HAD Reservoir relative to that from the GERD Reservoir. If the GERD Reservoir is filled quickly, the filling period will end sooner, and storage in the HAD Reservoir can begin to recover earlier. Furthermore, a longer filling period exposes this process to a longer duration that could be helpful or harmful, since it becomes increasingly likely that a high flood or a major drought might facilitate filling or increase system risks, respectively. We emphasize that there is a tradeoff between filling quickly versus filling slowly, in other words, causing greater immediate risks with a rapid decline of the HAD

Reservoir versus allowing more time for the GERD to fill, which prolongs low-level risks to the HAD Reservoir and Egyptian water supplies.

A third factor that will significantly influence the downstream impacts of the GERD during the filling process will be how rapidly the power generated by the GERD can be integrated into the regional power grid. Insufficient power demands can limit the ability of the GERD turbines to discharge water downstream, leaving releases to be limited by the hydraulic capacity of bottom outlets (542.25 m). A temporary 'low block' spillway will be used during at least the first two years of the filling process, which will be elevated each year to allow the reservoir capture additional water and establish a maximum water level for each year it is used [18]. The specific details of the turbine installation timing, the construction specifications of temporary spillways, and the expansion rate of the power grid are currently unknown. However, Ethiopia's ambitious Growth and Transformation Plan (GTP II) indicated an expansion of 4500 km of transmission lines between 2010 to 2015, a rate that is expected to continue in the coming years. Power sharing agreements with neighboring countries are also expected to play a significant role in the ability and willingness of Ethiopia to generate energy from the GERD, possibly reinforced by commitments of prioritization to downstream riparians.

A fourth factor particularly affecting the downstream impacts on Egypt will be how Sudan alters its irrigation withdrawals in response to the new flow regime during the filling period. Because the GERD's releases will reduce the monthly fluctuations in the Blue Nile flows, once the reservoir fills, Sudan will have an increase water supply during the crucial summer season when crop water requirements are high. This should enable Sudan to increase its irrigation withdrawals. Due to uncertainties in current withdrawals, a lack of agreement about how much additional could be diverted, and how rapidly Sudan might expand its irrigation use, this study does not include any

increased water withdrawals by Sudan due to increased availability of summer flows. Whether or not an increase in water withdrawals by Sudan could occur during the filling process would be a topic of negotiation, particularly between Sudan and Egypt.

A fifth important element that will significantly influence the impact of the GERD on Egypt during the filling process is the volume of water in the HAD Reservoir when the filling begins. This volume will act as an initial buffer to the volume retained by the GERD Reservoir, as of 24 August 2020, the HAD storage volume was nearly full at 178.4 masl, corresponding to a storage of 140 bcm including 7 bcm of sediment. Supplementary Figure 3 shows the estimated storage of the HAD based on satellite imagery (https://ipad.fas.usda.gov/cropexplorer/global_reservoir/).

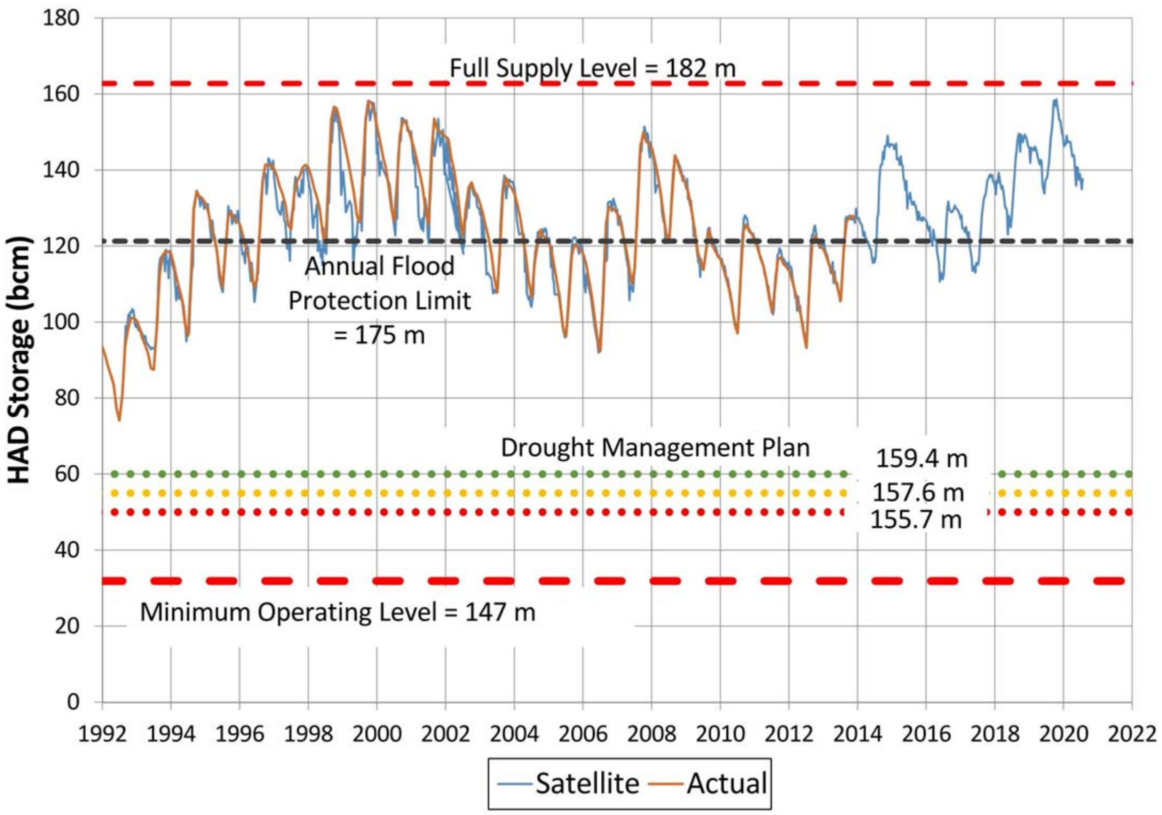

Supplementary Figure 3: HAD Storage levels by satellite imagery

A final factor affecting the downstream consequences of filling the GERD Reservoir is the operating policy of the HAD. Given the inevitability of declines in the elevation of the HAD Reservoir during filling, Egypt will have to continuously evaluate whether to continue to release 55.5 bcm annually and risk storage falling to the minimum operating level of 147 masl, or to proactively reduce releases from the HAD to lessen this risk. If the reservoir reaches this critical elevation, forced curtailments would result. A proactive approach implies earlier adverse impacts on Egyptian water users, but reduced risk of severe and especially damaging shortages. The current Drought Management Policy of the HAD is one form of a proactive plan to minimize risks. It predates the GERD, however, and therefore should be revisited given the impending changes to seasonal inflow patterns [4].

We also emphasize that Sudan, unlike Egypt, has no over-year water storage to buffer reduced upstream flow, so the filling and operation of GERD may cause concern if the intra-annual timing of releases is not known. With coordination between the GERD and the Sudanese dams and sufficient flows released from the GERD to meet current Sudanese irrigation withdrawals, the GERD will provide Sudan with a wide range of economic benefits, including flood control, increased hydropower from more stable water levels at dams in Sudan, increased summer water supply, and reduced sedimentation of its storage facilities [11,19,20] . These risks and benefits will occur immediately when filling begins and continue into the foreseeable future. Sudan is also likely to be a major market for the power that will be produced from the GERD and could eventually provide interconnections to Egypt as well.

<u>Supplementary Note 4: Evaporation considerations</u>

During the filling of the GERD Reservoir, the HAD Reservoir will operate at considerably lower levels, such that there will be modest declines in overall system evaporation. After the filling of the

GERD Reservoir, system evaporation will only remain lower so long as levels in the HAD Reservoir remain at considerably lower levels than in the pre-GERD era, which will only occur if the basin enters a period of drought and low flows, and/or if upstream abstractions of water increase. Thus, evaporative savings are greatest when downstream water scarcity is highest. Over the long term and assuming current levels of upstream water use, the addition of the GERD, with the substantial surface area of its reservoir, will increase the net evaporation of the system. After the GERD Reservoir filling is complete and the HAD Reservoir has recovered, the total additional evaporation from the GERD Reservoir will be 1.7 bcm per year, and the evaporation savings from a lower HAD Reservoir will be 1.1 bcm. The GERD Reservoir is assumed to have an annual average evaporation rate of 108 cm/year, compared to the HAD reservoir and Sudan's Merowe reservoir with 270 cm/year and 275 cm/year, respectively.

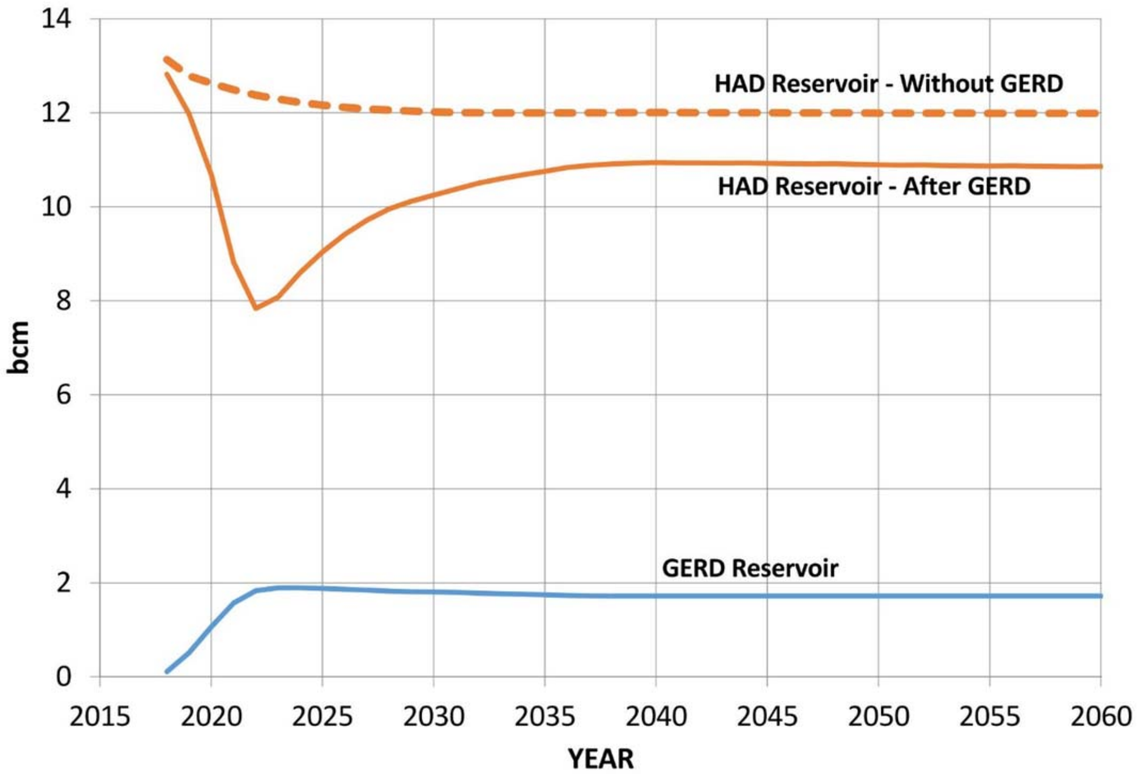

Supplementary Figure 4: Average GERD Reservoir and HAD Reservoir evaporation during filling and *new normal* conditions

Although this study emphasized the use of historical hydrologic scenarios to develop the narratives of the three eras, we also recognize the implications that climate change is likely to have on the Nile Basin. Various Global Climate Models (GCMs) tend to show a wide variation of projections of precipitation; however, most models tend to agree that increased temperatures are likely to occur[21,22]. Higher temperatures not only increase water consumption due to increased evapotranspiration[23], but also increase the relative advantage of storing water in less arid regions of the basin to minimize evaporative losses[24], thus increasing the relative benefit of storing water in the GERD Reservoir as compared to the HAD Reservoir.

Supplementary Note 5: Statistical trace selections

The selection of traces was based on an analysis of naturalized inflows to the HAD Reservoir from 1900 to 2018 [25,26]. This selection used both parametric and non-parametric criteria, including a statistical analysis of periods of flows along with hand selection to avoid anomalous events such as single-year floods or droughts. Instead, high, average and low samples of 10-year average flows used for the Era 1 analysis were chosen to include persistent extreme periods (high and low), and average conditions. A 20-year average trace was selected for Era 2. A 20-year drought trace was selected for entering a drought in Era 3, which was followed by a historically wet 10-year period.

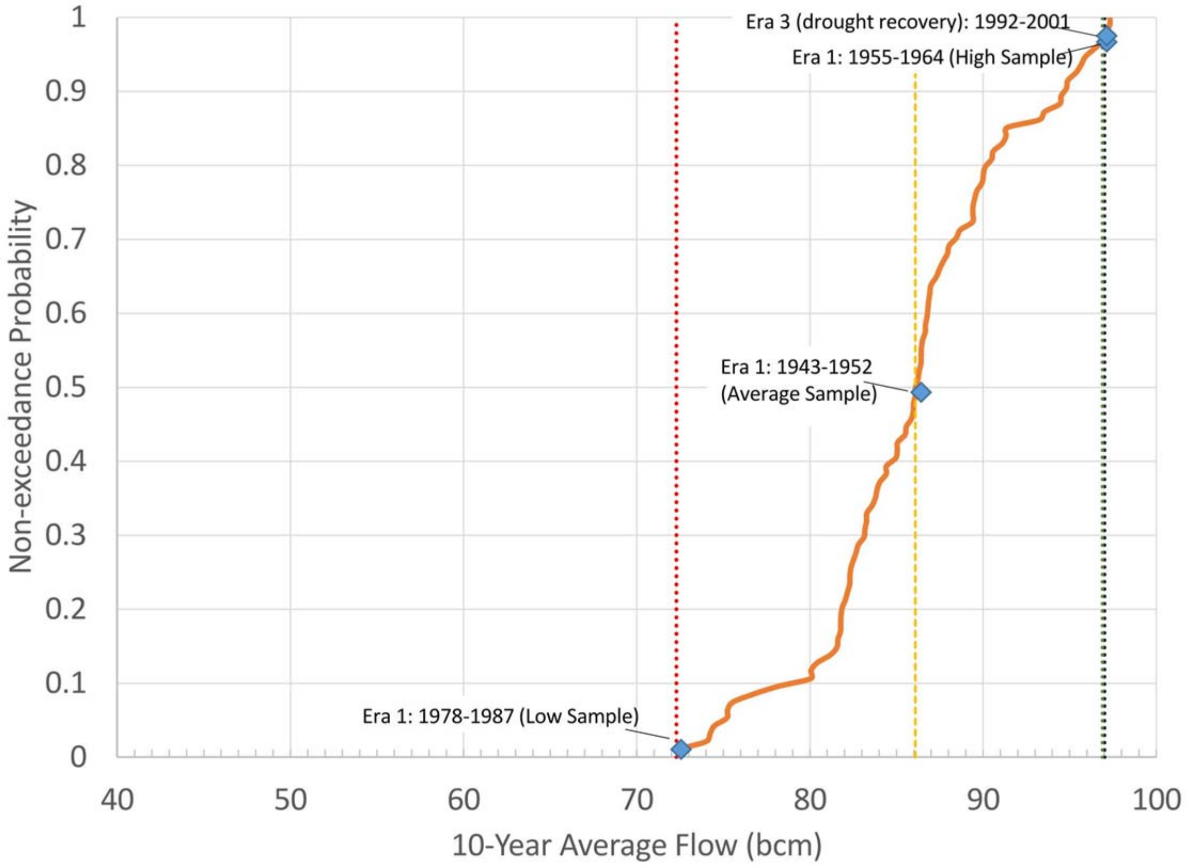

Supplementary Figure 5: Statistical distribution of 10-year average flows and selected traces for Era 1 and 3

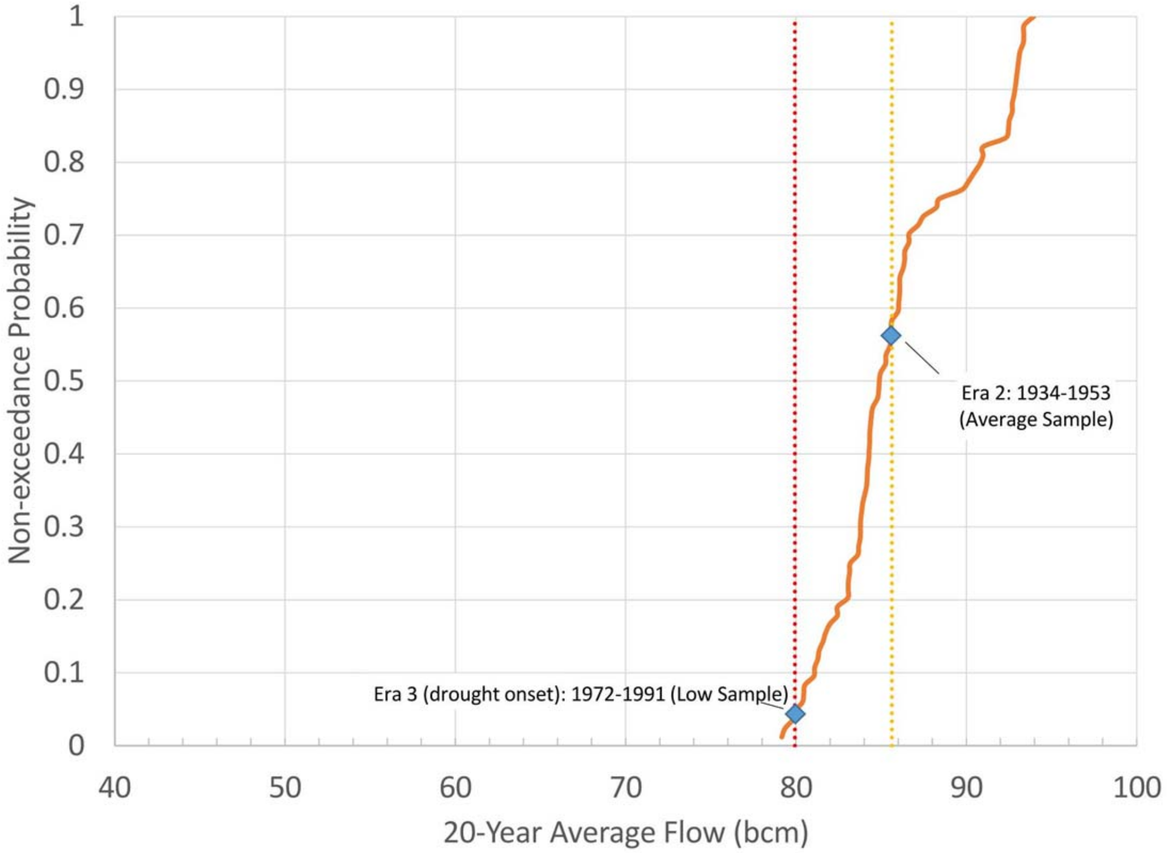

Supplementary Figure 6: Statistical distribution of 20-year average flows and selected traces for Era 2 and 3

<u>Supplementary Note 6: Sensitivity analysis of existing consumptive uses</u>

Given the uncertainty and controversy over current water abstractions for consumptive uses in Sudan, the implications of assumptions about these existing water withdrawals were examined during Era 1. Impacts on the HAD were evaluated assuming that total current withdrawals range from 12.2 to 16.7 bcm. Supplementary Figure 7 demonstrates the implications for HAD storage during Era 1 (filling) for the period beginning in 1972.

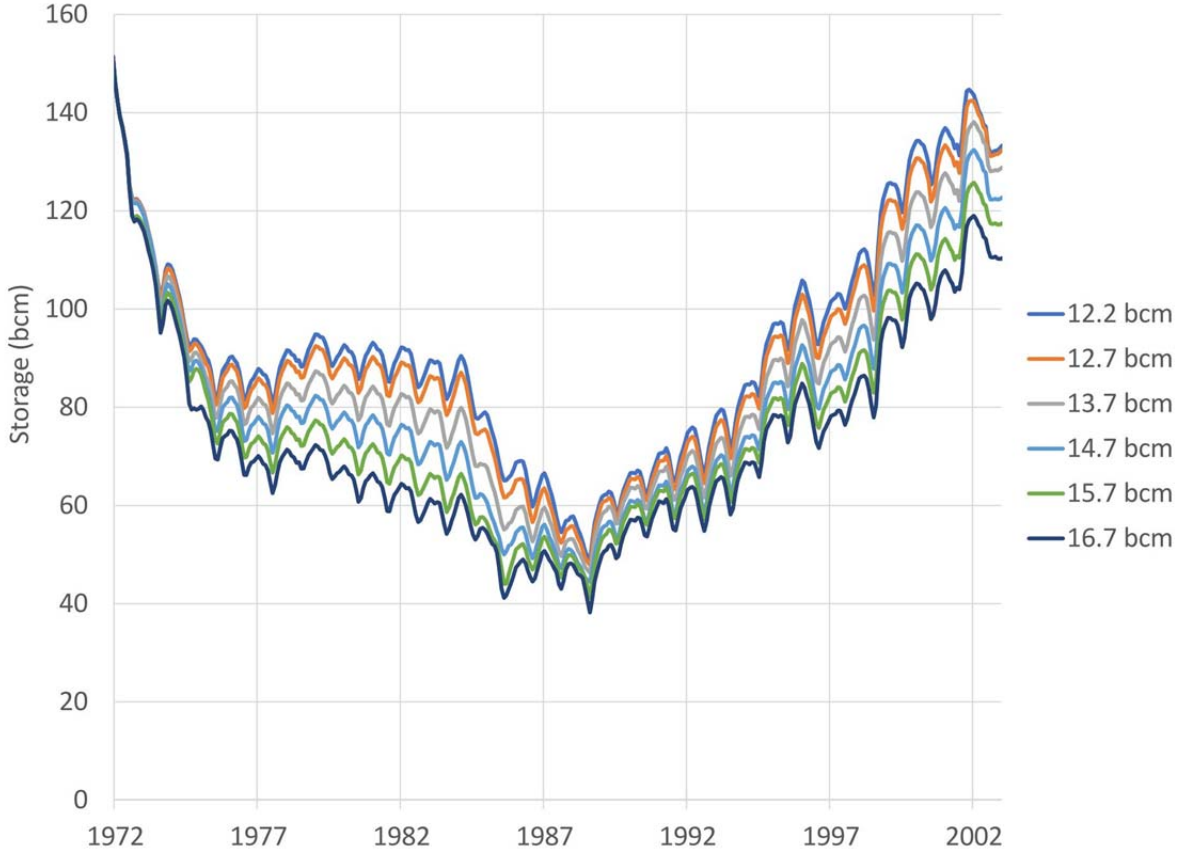

Supplementary Figure 7: Storage in the HAD Reservoir under different Sudanese depletion assumptions

Supplementary Table 1. Key elevations and storage values of modeled reservoirs

	Min Active Pool Elevation (m)	Max Supply Level (m)	Min Diversion Elevation (m)	Max Capacity (MW)	Active Storage Volume (MCM)	Total Storage Volume (MCM)
Tana/Beles	1783.5	1787.5	1783.5	423	12328	41080
Finchaa	2214.0	2219.0	--	134	1050	1120
GERD	590.0	640.0	--	6450	59000	74000
Roseries	471.0	490.0	--	280	5885	5909
Sennar	417.2	421.7	417.2	15	420	640
Tekeze	1096.0	1140.0	--	300	5289	9293
Upper Atbara/Setit	509.0	521.0	--	320	2508	3688
Khashm El Girba	463.0	474.0	463.5	10.6 + 7.6	597	657
Jebel Aulia	372.0	377.4	372.0	28.8	3065	3290
Merowe	285.0	300.0	--	1250	8182	12396
High Aswan	147.0	182.0	178.0	2100	148040	182700*

* HAD storage reduced by 7 bcm due to sedimentation

Supplementary Table 2. Key modeling assumptions for the analysis of three stylized eras

Country-specific assumptions	Egypt	Sudan	Ethiopia
Water demand during filling (bcm/yr)	55.5	16.7*	0.45**
Future water demand (bcm/yr)	55.5	16.7*	1.45**
Infrastructure operations	HAD release: 55.5 bcm/yr (incl. 4 bcm/yr pumping for the Toshka project), flood control space created by Aug 1, demands adjusted w/DMP.***	Roseries: Releases for Blue Nile agriculture and min channel flows<br>Sennar: Direct diversion for Gezira/Managil, releases for min channel flows<br>Merowe: Releases for monthly power generation and min channel flows	GERD operates for firm energy generation equivalent to 1600 MW continuous output with 90% reliability.****
GERD filling	n.a.	n.a.	Minimum release: 35 bcm/yr

* Total measured from the point of diversion.
** In addition to the irrigation withdrawals around Lake Tana, which are not explicitly modeled.
*** DMP is the Drought Management Policy currently used by Egypt (see Supplementary Note 1 above).
**** The objective is to generate *firm energy*. With the 5150 MW installed capacity of the GERD, it would be possible to generate energy in a very different non-firm pattern.

Supplementary Table 3: Naturalized average annual flow at Aswan (bcm/yr) and years of hydrology

	Dry	Average	Wet
Era 1 - Filling	72.3 (1978-1987)	86.1 (1943-1952)	96.9 (1955-1964)
Era 2 – New Normal	--	85.6 (1934-1953)	--
Era 3 – Multi-year Drought*	Drought onset: 79.9 (1972-1991)	--	Drought recovery: 97.0 (1992-2001)

* The lowest cumulative flow period over 20 years was used to select the multi-year drought period, and this was followed by a 10-year recovery period for the recovery. In the chosen sequence, however, the drought subsides in the 17th year and recovery therefore occurs over the subsequent 13 years.

Supplementary Table 4. Effects of the GERD filling across low, average and high hydrologic scenarios

	Low Flow Sequence 1978-1987		Average Flow Sequence 1943-1952		High Flow Sequence 1955-1964	
	w/o GERD	with GERD	w/o GERD	with GERD	w/o GERD	with GERD
**Ethiopia**						
Average annual GERD hydropower generation (TWh)	--	8.13	--	11.56	--	12.47
Average annual GERD evaporation (bcm)	--	1.2	--	1.5	--	1.5
Ending GERD Storage (bcm)	--	43.6	--	68.4	--	72.0
**Sudan**						
Average annual hydropower generation* (TWh)	7.59	8.21	8.57	9.39	9.24	9.85
Average annual evaporation* (bcm)	2.8	3.1	2.9	3.3	3.0	3.3
*includes Merowe + Roseries + Sennar						
**Egypt**						
Average annual HAD hydropower generation (TWh)	7.04	6.13	8.62	7.39	9.01	8.28
Average annual HAD evaporation (bcm)	10.2	8.3	14.2	10.8	15.2	13.2
Cumulative deficit (bcm)	21.9	49.1	0.0	0.0	0.0	0.0
No. of years with deficit	4	6	0	0	0	0
Ending HAD Reservoir storage (bcm)	41.7	37.0	117.0	77.7	151.3	144.8

Supplementary Table 5. Effects of the GERD during *New Normal* operations under a selection of average hydrologic conditions

	Average Flow Sequence 1927-1946		Average Flow Sequence 1934-1953		Average Flow Sequence 1939-1958	
	w/o GERD	with GERD	w/o GERD	with GERD	w/o GERD	with GERD
**Ethiopia**						
Average annual GERD hydropower generation (TWh)	--	15.71	--	15.66	--	15.55
Average annual GERD evaporation (bcm)	--	1.8	--	1.8	--	1.8
Ending GERD Storage (bcm)	--	71.2	--	70.4	--	71.6
**Sudan**						
Average annual hydropower generation* (TWh)	8.41	10.43	8.53	10.51	8.56	10.51
Average annual evaporation* (bcm)	2.9	3.5	2.9	3.5	2.9	3.5
*includes Merowe + Roseries + Sennar						
**Egypt**						
Average annual HAD hydropower generation (TWh)	7.42	7.13	7.55	7.20	7.19	6.81
Average annual HAD evaporation (bcm)	10.9	10.2	11.2	10.4	10.4	9.4
Cumulative deficit (bcm)	1.2	0.3	0	0	18.0	14.5
No. of years with deficit	1	1	2	0	7	6
Ending HAD Reservoir storage (bcm)	106.2	82.1	100.0	78.7	134.7	113.4

Supplementary Table 6. Effects of the GERD during the onset and recovery from drought

	Low Flow Sequence 1972-1987		High Flow Sequence 1988-2001	
	w/o GERD	with GERD	w/o GERD	with GERD
**Ethiopia**				
Average annual GERD hydropower generation (TWh)	--	12.98	--	14.02
Average annual GERD evaporation (bcm)	--	1.5	--	1.6
Ending GERD Storage (bcm)	--	18.4	--	71.6
**Sudan**				
Average annual hydropower generation* (TWh)	8.01	10.00	8.84	10.56
Average annual evaporation* (bcm)	2.9	3.4	3.0	3.5
*includes Merowe + Roseries + Sennar				
**Egypt**				
Average annual HAD hydropower generation (TWh)	6.28	6.31	7.51	7.04
Average annual HAD evaporation (bcm)	8.3	8.3	11.2	10.0
Cumulative deficit (bcm)	42.2	27.3	14.2	21.4
No. of years with deficit	8	6	5	6
Ending HAD Reservoir storage (bcm)	39.3	48.5	142.9	118.6

Supplementary References

1 Arjoon, D., Mohamed, Y., Goor, Q. & Tilmant, A. Hydro-economic risk assessment in the eastern Nile River basin. *Water Resources and Economics* **8**, 16-31, doi:10.1016/j.wre.2014.10.004 (2014).

2 Goor, Q., Halleux, C., Mohamed, Y. & Tilmant, A. Optimal operation of a multipurpose multireservoir system in the Eastern Nile River Basin. *Hydrol. Earth Syst. Sci.* **14**, 1895-1908, doi:10.5194/hess-14-1895-2010 (2010).

3 Arjoon, D., Tilmant, A. & Herrmann, M. Sharing water and benefits in transboundary river basins. *Hydrol. Earth Syst. Sci.* **20**, 2135-2150, doi:10.5194/hess-20-2135-2016 (2016).

4 Wheeler, K. G. *et al.* Exploring Cooperative Transboundary River Management Strategies for the Eastern Nile Basin. *Water Resources Research* **54**, 9224-9254, doi:doi:10.1029/2017WR022149 (2018).

5 Block, P. J. & Strzepek, K. Economic analysis of large-scale upstream river basin development on the Blue Nile in Ethiopia considering transient conditions, climate variability, and climate change. *Journal of Water Resources Planning and Management* **136**, 156-166, doi:10.1061/(ASCE)WR.1943-5452.0000022 (2010).

6 King, A. & Block, P. An assessment of reservoir filling policies for the Grand Ethiopian Renaissance Dam. *Journal of Water and Climate Change* **5**, 233-243, doi:10.2166/wcc.2014.043 (2014).

7 Zhang, Y., Block, P., Hammond, M. & King, A. Ethiopia's Grand Renaissance Dam: Implications for Downstream Riparian Countries. *Journal of Water Resources Planning and Management*, doi:10.1061/(ASCE)WR.1943-5452.0000520 (2015).

8 Zhang, Y., Erkyihum, S. T. & Block, P. Filling the GERD: evaluating hydroclimatic variability and impoundment strategies for Blue Nile riparian countries. *Water International* **41**, 593-610, doi:10.1080/02508060.2016.1178467 (2016).

9 Jeuland, M. Economic implications of climate change for infrastructure planning in transboundary water systems: An example from the Blue Nile. *Water Resources Research* **46**, W11556, doi:10.1029/2010WR009428 (2010).

10 Jeuland, M. & Whittington, D. Water resources planning under climate change: Assessing the robustness of real options for the Blue Nile. *Water Resources Research* **50**, 2086-2107, doi:10.1002/2013WR013705 (2014).

11 Jeuland, M., Wu, X. & Whittington, D. Infrastructure development and the economics of cooperation in the Eastern Nile. *Water International*, 1-21, doi:10.1080/02508060.2017.1278577 (2017).

12 Nigatu, G. & Dinar, A. Economic and hydrological impacts of the Grand Ethiopian Renaissance Dam on the Eastern Nile River Basin. *Environment and Development Economics* **21**, 532-555, doi:10.1017/S1355770X15000352 (2015).

13 Kahsay, T. N. *et al.* A hybrid partial and general equilibrium modeling approach to assess the hydro-economic impacts of large dams – The case of the Grand Ethiopian Renaissance Dam in the Eastern Nile River basin. *Environmental Modelling & Software* **117**, 76-88, doi:https://doi.org/10.1016/j.envsoft.2019.03.007 (2019).

14 Borgomeo, E., Mortazavi-Naeini, M., Hall, J. W. & Guillod, B. P. Risk, Robustness and Water Resources Planning Under Uncertainty. *Earth's Future* **6**, 468-487, doi:10.1002/2017ef000730 (2018).

15 Borgomeo, E., Mortazavi-Naeini, M., Hall, J. W., O'Sullivan, M. J. & Watson, T. Trading-off tolerable risk with climate change adaptation costs in water supply systems. *Water Resources Research* **52**, 622-643, doi:10.1002/2015WR018164 (2016).

16 Wheeler, K. G. *et al.* Cooperative filling approaches for the Grand Ethiopian Renaissance Dam. *Water International* **41**, 611-634, doi:10.1080/02508060.2016.1177698 (2016).

17 Blackmore, D. & Whittington, D. Opportunities for cooperative water resources development on the eastern Nile: Risks and rewards, Final Report - An Independent Report of the Scoping Study Team to the Eastern Nile Council of Ministers. (Washington D.C., 2008).
18 Basheer, M. *et al.* Filling Africa's largest hydropower dam should consider engineering realities. *One Earth* (In Print), *doi.org/10.1016/j.oneear.2020.08.015* (2020).
19 Basheer, M. & Ahmed Elagib, N. Temporal analysis of water-energy nexus indicators for hydropower generation and water pumping in the Lower Blue Nile Basin. *Journal of Hydrology* **578**, 124085, doi:10.1016/j.jhydrol.2019.124085 (2019).
20 Basheer, M. *et al.* Quantifying and evaluating the impacts of cooperation in transboundary river basins on the Water-Energy-Food nexus: The Blue Nile Basin. *Science of The Total Environment* **630**, 1309-1323, doi:10.1016/j.scitotenv.2018.02.249 (2018).
21 Conway, D. From headwater tributaries to international river: Observing and adapting to climate variability and change in the Nile basin. *Global Environmental Change* **15**, 99-114, doi:http://dx.doi.org/10.1016/j.gloenvcha.2005.01.003 (2005).
22 Conway, D. Water resources: Future Nile river flows. *Nature Clim. Change* **7**, 319-320, doi:10.1038/nclimate3285 (2017).
23 Hasan, E., Tarhule, A., Kirstetter, P.-E., Clark, R. & Hong, Y. Runoff sensitivity to climate change in the Nile River Basin. *Journal of Hydrology* **561**, 312-321, doi:https://doi.org/10.1016/j.jhydrol.2018.04.004 (2018).
24 Wang, W. *et al.* Global lake evaporation accelerated by changes in surface energy allocation in a warmer climate. *Nature Geoscience* **11**, 410-414, doi:10.1038/s41561-018-0114-8 (2018).
25 van der Krogt, W. & Ogink, H. Development of the Eastern Nile Water Simulation Model. Report No. 1206020-000-VEB-0010, (Deltares, Delft, 2013).
26 Sudan MoIWR. Gage Flows at Dongola Station (Sudan Ministry of Irrigation and Water Resources, 2019).
